# Prevalence and temporal relationship of clinical co-morbidities in idiopathic dystonia: a UK linkage-based study

**DOI:** 10.1007/s00415-024-12284-6

**Published:** 2024-03-21

**Authors:** Grace A. Bailey, Anna Rawlings, Fatemeh Torabi, W. Owen Pickrell, Kathryn J. Peall

**Affiliations:** 1 Neuroscience and Mental Health Research Institute, Division of Psychological Medicine and Clinical Neurosciences, Cardiff University School of Medicine, Hadyn Ellis Building, Maindy Road, Cardiff, CF24 4HQ UK; 2 Swansea University Medical School, Singleton Park, Swansea, UK; 3 Department of Neurology, Morriston Hospital, Swansea Bay University Health Board, Swansea, UK; 4 Health Data Research UK, Swansea, UK

**Keywords:** Dystonia, Co-morbidity, Epidemiology, Linked clinical data

## Abstract

While motor and psychiatric phenotypes in idiopathic dystonia are increasingly well understood, a few studies have examined the rate, type, and temporal pattern of other clinical co-morbidities in dystonia. Here, we determine the rates of clinical diagnoses across 13 broad systems-based diagnostic groups, comparing an overall idiopathic dystonia cohort, and sub-cohorts of cervical dystonia, blepharospasm, and dystonic tremor, to a matched-control cohort. Using the SAIL databank, we undertook a longitudinal population-based cohort study (January 1st 1994–December 31st 2017) using anonymised electronic healthcare records for individuals living in Wales (UK), identifying those diagnosed with dystonia through use of a previously validated algorithm. Clinical co-morbid diagnoses were identified from primary health care records, with a 10% prevalence threshold required for onward analysis. Using this approach, 54,166 dystonia cases were identified together with 216,574 matched controls. Within this cohort, ten of the main ICD-10 diagnostic codes exceeded the 10% prevalence threshold over the 20-year period (infection, neurological, respiratory, gastrointestinal, genitourinary, dermatological, musculoskeletal, circulatory, neoplastic, and endocrinological). In the overall dystonia cohort, musculoskeletal (aOR: 1.89, aHR: 1.74), respiratory (aOR: 1.84; aHR: 1.65), and gastrointestinal (aOR: 1.72; aHR: 1.6) disorders had the strongest associations both pre- and post-dystonia diagnosis. However, variation in the rate of association of individual clinical co-morbidities was observed across the cervical, blepharospasm, and tremor dystonia groups. This study suggests an increased rate of specific co-morbid clinical disorders both pre- and post-dystonia diagnosis which should be considered during clinical assessment of those with dystonia to enable optimum symptomatic management.

**Supplementary Information:**

The online version contains supplementary material available at 10.1007/s00415-024-12284-6.

## Introduction

Dystonia is a hyperkinetic movement disorder characterised by abnormal movements or postures [1], with an estimated prevalence of 120/100,000 [2]. Recent work has highlighted the co-morbid non-motor symptom profile of dystonia, focusing predominantly on psychiatric, cognitive, pain, and sleep symptoms [3]. However, little work to date has examined whether those diagnosed with dystonia are at increased risk of other medical disorders, potentially complicating treatment options and care needs, as well as increasing healthcare utilisation and overall burden of disease.

Although dystonia does not reduce life expectancy [2], poor health-related quality of life is consistently reported [4, 5]. The few studies to date investigating dystonia co-morbidities have had varying results. Some have suggested higher rates of thyroid dysfunction [6–8] and cardiovascular changes, including heart rate and blood pressure variability [9]. Respiratory symptoms have also been recognised, including stridor, dyspnoea, and gasping [10, 11]. While a more recent population-based study of cervical dystonia found higher rates of cervical spondylosis, soft-tissue disorders, back pain, essential tremor, neck tension, dental caries, and abdominal and pelvic pain, compared to a control cohort [12]. Autoimmune disorders, such as thyroid disease [13], myasthenia gravis [14], and Sjogren’s syndrome [15], have also been reported at higher rates, with preliminary mixed immunoassays potentially indicating common autoimmune mechanisms in dystonia [16]. Respiratory symptoms have been attributed to dystonic spasms affecting the respiratory muscles, with evidence of upper airway or diaphragmatic dysfunction [17, 18].

To date, a few limited studies have examined the nature and rate of medical co-morbidities in dystonia, and none have examined their temporal relationship to dystonia motor symptom onset. Here, we examine a previously identified dystonia and matched-control population cohort with linked clinical data. This work determines those medical co-morbidities present at higher rates amongst those with dystonia, and their temporal pattern relative to dystonia motor symptom onset. Awareness of whether medical co-morbidities are at an excess in dystonia is key in developing more comprehensive clinical care pathways as well as potentially aiding in determining underlying causative mechanisms.

## Methods

### Study design and data sources

Using both retrospective and prospective analyses, the SAIL Databank (Swansea University, UK: www.saildatabank.com) was used to investigate the rate of medical co-morbidities in individuals diagnosed with dystonia. SAIL is an anonymised data repository containing health, education, and social care data covering the population of Wales. Clinical data are available from multiple sources, including primary (general practice) and secondary (hospital) care records, with data available for 86% and 100%, respectively, of the Welsh population (3.1 million).

### Dystonia diagnosis

Derivation and diagnostic validation of the dystonia cohort has been described elsewhere [2]. In brief, a reference population of 90 patients with a clinically confirmed diagnosis of adult-onset idiopathic focal cervical dystonia (AOIFCD) was linked to records in SAIL. Codes relevant to dystonia [Read code-primary care or International Classification of Diseases version 10 (ICD-10) codes-secondary care] were reviewed and used to identify 54,966 individuals with idiopathic dystonia throughout the study period (January 1994–December 2017).

### Study cohort

The dystonia cohort consisted of 54,166 individuals diagnosed with idiopathic dystonia (800 had no matched controls and were therefore not included) and 216,574 controls matched on: year of birth, sex, year of study entry, year of follow-up, and deprivation index quintile, their derivation has been described elsewhere [2]. Controls were randomly matched on a 1:4 (case:control, *n* = 54,121, 99.9%) basis using individuals with Welsh Longitudinal General Practice (WLGP) records, the remaining 45 cases were matched on a ratio of 1:1–3. Individuals were required to be resident in Wales at the time of index date and have an age, sex, and GP registration date recorded. Controls were assigned the same index date as their matched cases.

### Comorbidities

We obtained data on medical co-morbidities using primary care (WLGP) datasets (January 1994 and December 2017). With the exception of mental disorders that have been explored elsewhere [19], we examined all medical diagnostic categories, including: infectious and parasitic diseases, neoplasms, endocrine, nutritional, metabolic and immunity disorders, diseases of blood and blood-forming organs, nervous system and sense organ diseases, circulatory system diseases, respiratory system diseases, digestive system diseases, genitourinary system diseases, complications of pregnancy, childbirth and the puerperium, skin and subcutaneous tissue diseases, musculoskeletal and connective tissue diseases, congenital abnormalities, and perinatal conditions (Supplementary Table 1). To ensure consistent data coverage, only diagnoses recorded 10 years before and after the index date were analysed.

### Statistical analysis

During the primary analyses, individuals having received ≥ 1 diagnosis of any disorder within the broader disease categories (main Read code diagnoses) were identified. Only those affecting a minimum of 10% of the dystonia cohort were further analysed to ensure clinical relevance and consistency with approaches used elsewhere (Table 1 and Supplementary Tables 3–5) [20]. Subsequently, a nested case–control design was used to examine the association of each co-morbidity and idiopathic dystonia. Logistic regression analysis was used to estimate the risk of developing these co-morbidities during the retrospective time periods, with odds ratios (ORs) and 95% confidence intervals (CIs) calculated and adjusted for contact with medical services and/or clinical professionals. Medical contact was defined as any general practice interaction (diagnosis, symptom, and diagnostic testing) or secondary care contact (in-patient hospital admission and out-patient appointment), with each contact considered an independent event. A cohort study design was used to determine rates of medical diagnoses following dystonia diagnosis, with only those not diagnosed with each co-morbidity at the index date included for onward analysis. Hazard ratios (HR) and 95% CIs for each co-morbidity were calculated using Cox proportional hazard regression, again adjusted for the total number of medical contacts. The follow-up period was censored at the first co-morbid diagnosis, death, and loss to follow-up or 10 years after the index date. Bonferroni correction was used to adjust p values for both retrospective and prospective methods.

**Table 1:** Frequency of co-morbid diagnoses within main diagnostic categories

Disease category	Total number of cases		Pre-dystonia diagnosis		Post-dystonia diagnosis	
	Dystonia (*n*, %) (*n* = 54,166)	Controls (*n*, %) (*n* = 216,574)	Dystonia (*n*, %) (*n* = 54,166)	Controls (*n*, %) (*n* = 216,574)	Dystonia (*n*, %) (*n* = 54,166)	Controls (*n*, %) (*n* = 216,574)
Infectious and parasitic diseases	**26,809 (49.9)**	**77,601 (35.8)**	**13,225 (24.42)**	**34,845 (16.09)**	**20,041 (37)**	**56,649 (26.2)**
Neoplasms	**10,308 (19)**	**32,829 (15.2)**	4022 (7.43)	11,599 (5.36)	**7532 (13.9)**	**24,509 (11.3)**
Endocrine, nutritional, metabolic, and immunity disorders	**9493 (17.5)**	**27,479 (12.7)**	3906 (7.21)	10,654 (4.92)	**7081 (13.1)**	20,536 (9.5)
Diseases of blood and blood-forming organs	3693 (6.8)	10,642 (4.9)	1428 (2.64)	3832 (1.77)	2545 (4.7)	7477 (3.45)
Nervous system and sense organ diseases	**32,578 (60.1)**	**97,175 (44.9)**	**16,798 (31.01)**	**45, 136 (20.84)**	**25,803 (47.5)**	**75,086 (34.7)**
Circulatory system diseases	**14,826 (27.4)**	**45,537 (21)**	**7,070 (13.05)**	19,852 (9.17)	**11,011 (20.3)**	**33,692 (15.6)**
Respiratory system diseases	**39,256 (72.5)**	**121,244 (56)**	**23,511 (43.41)**	**62,815 (29)**	**32,966 (60.9)**	**98,051 (45.3)**
Digestive system diseases	**23,181 (42.8)**	**62,858 (29)**	**10,513 (19.41)**	**25,922 (11.97)**	**17,857 (33)**	**47,280 (21.8)**
Genitourinary system diseases	**24,411 (45.1)**	**73,045 (33.7)**	**11,557 (21.34)**	**31,181 (14.4)**	**19,386 (35.8)**	**57,020 (26.3)**
Complications of pregnancy, childbirth, and the puerperium	4546 (8.4)	16,333 (7.5)	1836 (3.39)	6587 (3.04)	3179 (5.9)	11,409 (5.3)
Skin and subcutaneous tissue diseases	**34,681 (64)**	**108,202 (50)**	**17,299 (31.94)**	**48,359 (22.33)**	**28,597 (52.8)**	**87,306 (40.3)**
Musculoskeletal and connective tissue	**35,756 (66)**	**101,594 (46.9)**	**18,410 (33.99)**	**44,933 (20.75)**	**30,735 (56.7)**	**84,362 (39)**
Congenital anomalies	1581 (2.9)	3949 (1.8)	704 (1.3)	1693 (0.78)	948 (1.8)	2424 (1.1)
Perinatal conditions	226 (0.4)	639 (0.3)	111 (0.2)	288 (0.13)	119 (0.2)	354 (0.2)

Bold values represent > 10% affected

### Ethics

This study design uses anonymized, routinely collected data, and therefore does not require ethical approval and written informed consent. The SAIL independent Information Governance Review Panel (IGRP), consisting of experts in information governance and members of the public, approved this study (Reference: 0768).

## Results

Using the previously described dystonia case ascertainment algorithm, 54,166 dystonia cases were identified together with 216,574 matched controls. Demographic characteristics are provided in Supplementary Table 2. Overall, ten primary ICD-10 codes exceeded the 10% prevalence threshold in both the dystonia and control cohorts over the 20-year period examined (Table 1). Evaluation of the individual dystonia diagnostic categories (cervical dystonia, blepharospasm, and dystonic tremor) identified similar patterns to the overall cohort but with differences observed in the number and type of co-morbid medical disorders observed pre-dystonia diagnosis (Supplementary Tables 3–5). Here, infectious, nervous, respiratory, gastrointestinal (GI), genitourinary, dermatological, and musculoskeletal disorders were observed in those with cervical dystonia (Supplementary Table 3), with the addition of circulatory disorders in the blepharospasm cohort (Supplementary Table 4), and neoplastic and endocrinological disorders in the tremor cohort (Supplementary Table 5). All primary ICD-10 diagnostic groups exceeding the 10% threshold were taken forward for analysis of their relative risk of diagnosis both pre- and post-dystonia diagnosis.

### Overall dystonia

Amongst the overall cohort (*n* = 54,166), musculoskeletal and connective tissue (aOR: [adjusted odds ratio): 1.89; aHR: [adjusted Hazard ratio]: 1.74), respiratory diseases (aOR: 1.84; aHR: 1.65), and GI system diseases (aOR: 1.72; aHR: 1.6) had the strongest associations both pre- and post-dystonia diagnosis (Table 2). Within each of these diagnostic groups, similar sub-diagnostic codes were noted to have the highest associations with exception of osteopathy/chondropathy/acquired musculoskeletal deformity pre-dystonia diagnosis and musculoskeletal and connective tissue diseases post-dystonia diagnosis in the musculoskeletal category. Each of the diagnoses demonstrated a higher relative risk of diagnosis prior to dystonia diagnosis, with exception of musculoskeletal and connective tissue diagnostic group where the higher risk was observed (aHR: 2.91, 95% CI 2.12–4.01) following dystonia diagnosis (Table 2).

**Table 2:** Adjusted Odds and Hazard Ratios for overall and cervical dystonia groups

Overall				Cervical dystonia			
Pre-dystonia [aOR, (95%CI), *p* value]		Post-dystonia [aHR, (95%CI), *p* value]		Pre-dystonia [aOR, (95%CI), *p* value]		Post-dystonia [aHR, (95%CI), *p* value]	
MSK	1.89 (1.85–1.93) < 2e−16	MSK	1.74 (1.71–1.77) < 2e−16	Respiratory	1.92 (1.87–1.96) < 2e−16	MSK	1.71 (1.68–1.74) < 2e−16
Respiratory	1.84 (1.8–1.88) < 2e−16	Respiratory	1.65 (1.62–1.68) < 2e−16	ID	1.75 (1.71–1.8) < 2e−16	Respiratory	1.7 (1.66–1.73) < 2e−16
GI	1.72 (1.68–1.77) < 2e−16	GI	1.6 (1.57–1.63) < 2e−16	MSK	1.74 (1.7–1.79) < 2e−16	ID	1.59 (1.56–1.63) < 2e−16
Nervous system	1.66 (1.63–1.7) < 2e−16	Nervous system	1.58 (1.55–1.61) < 2e−16	Nervous system	1.6 (1.57–1.65) < 2e−16	nervous system	1.53 (1.5–1.56) < 2e−16
Infectious	1.66 (1.62–1.7) < 2e−16	Infectious	1.56 (1.53–1.59) < 2e−16	Skin	1.56 (1.52–1.6) < 2e−16	Skin	1.48 (1.45–1.51) < 2e−16
Skin	1.59 (1.55–1.62) < 2e−16	Skin	1.51 (1.49–1.54) < 2e−16	GU	1.54 (1.5–1.59) < 2e−16	GI	1.47 (1.44–1.5) < 2e−16
GU	1.57 (1.53–1.61) < 2e−16	GU	1.42 (1.4–1.45) < 2e−16	GI	1.52 (1.48–1.57) < 2e−16	GU	1.4 (1.37–1.43) < 2e−16
Circulation	1.44 (1.39–1.48) < 2e−16	Endocrine	1.35 (1.31–1.39) < 2e−16			Neoplasm	1.12 (1.08–1.16) 1.04e−11
		Neoplastic	1.21 (1.17–1.24) < 2e−16			Endocrine	1.08 (1.04–1.1) 5.42e−05
		Circulation	1.09 (1.06–1.11) < 2e−16				

Table only includes disorders where statistically significant (*p* < 0.001) difference was observed between dystonia and control cohorts

*aHR* adjusted hazard ratio, *aOR* adjusted odds ratio, *95% CI* 95% confidence interval, *GI* gastrointestinal system, *GU* genitourinary system, *ID* infectious diseases, *MSK* musculoskeletal system

### Cervical dystonia

Of those diagnosed with cervical dystonia (*n* = 36,846), the strongest associations were seen across the same three primary ICD-10 diagnostic categories—respiratory, infectious, and musculoskeletal disorders—both pre- and post-dystonia diagnosis, with adjusted odds ratios being higher than their adjusted hazard ratio counterpart (Table 2). Examining individual secondary diagnoses (Table 4b), the highest adjusted odds ratios (pre-dystonia) were observed for poliomyelitis and other non-arthropod borne diseases [aOR: 3.02 (95% CI 1.61–5.44)] and vertebral column syndromes [aOR: 2.04 (95% CI 1.97–2.11)], with musculoskeletal and connective tissue diseases [aHR: 3.43 (95% CI 2.45–4.78)] and vertebral column syndromes [(aHR: 2.04 (95% CI 1.99–2.09)] demonstrating the highest association in the post-dystonia diagnostic period.

### Blepharospasm

Within the blepharospasm cohort (*n* = 1291), both nervous system disorders [aOR: 2.32 (95% CI 2.07–2.59); aHR: 1.73 (95% CI 1.67–1.78)] and musculoskeletal disorders [aOR: 2.06 (95% CI 1.83–2.31); aHR: 1.72 (95% CI 1.55–1.9)] demonstrated the strongest associations before and after dystonia diagnosis. Skin and GI disorders formed the next group of disorders, demonstrating a higher association post-blepharospasm diagnosis compared to pre-diagnosis (Table 3). More specifically, disorders involving the peripheral nervous system were the most highly associated with a diagnosis of blepharospasm both before [aOR: 4.98 (95% CI 3.87–6.3)] and after [aHR: 3.12 (95% CI 2.49–3.87)] dystonia diagnosis. Other more strongly associated disorders included those of the eye and adnexa [aOR: 2.73 (95% CI 2.4–3.11)] and disorders involving the upper GI tract (oesophagus, stomach, and duodenum) [aHR: 2.05 (95% CI 1.76–2.4)] (Table 4C).

**Table 3:** Adjusted Odds and Hazard Ratios for blepharospasm and dystonic tremor groups

Blepharospasm				Dystonic tremor			
Pre-dystonia [aOR, (95%CI), *p* value]		Post-dystonia [aHR, (95%CI), *p* value]		Pre-dystonia [aOR, (95%CI), *p* value]		Post-dystonia [aHR, (95%CI), *p* value]	
Nervous system	2.32 (2.07–2.59) < 2e−16	Nervous System	1.73 (1.67–1.78) < 2e−16	Circulation	2.48 (2.4–2.59) < 2e−16	GI	2.04 (1.97–2.12) < 2e−16
MSK	2.06 (1.83–2.31) < 2e−16	MSK	1.72 (1.55–1.9) < 2e−16	Endocrine	2.32 (2.2–2.45) < 2e−16	MSK	1.89 (1.83–1.95) < 2e−16
Skin	1.86 (1.66–2.08) < 2e−16	GI	1.64 (1.47–1.84) < 2e−16	MSK	2.25 (2.17–2.33) < 2e−16	Nervous system	1.73 (1.67–1.78) < 2e−16
GI	1.81 (1.57–2.07) < 2e−16	Skin	1.48 (1.33–1.64) 6.74E−-14	GI	2.22 (2.14–2.31) < 2e−16	Skin	1.65 (1.6–1.7) < 2e−16
Circulation	1.74 (1.49–2.02) 1e−12	Neoplasm	1.44 (1.23–1.67) 2.87E−06	Nervous System	1.76 (1.7–1.82) < 2e−16	Respiratory	1.61 (1.6–1.66) < 2e−16
Respiratory	1.71 (1.53–1.91) < 2e−16	GU	1.38 (1.23–1.55) 3.79E−08	Respiratory	1.7 (1.65–1.76) < 2e−16	GU	1.52 (1.46–1.57) < 2e−16
GU	1.64 (1.44–1.87) 1.96E−13	Resp.	1.3 (1.17–1.45) 2.36E−06	Skin	1.66 (1.61–1.72) < 2e−16	Endocrine	1.51 (1.44–1.58) < 2e−16
Infectious	1.51 (1.32–1.72) 6.78E−10			GU	1.65 (1.59–1.72) < 2e−16	Infectious	1.5 (1.45–1.56) < 2e−16
				Infectious	1.48 (1.42–1.54) < 2e−16	Circulation	1.36 (1.31–1.42) < 2e−16

Table only includes disorders where statistically significant (*p* < 0.001) difference was observed between dystonia and control cohorts

*aHR* adjusted hazard ratio, *aOR* adjusted odds ratio, *95% CI* 95% Confidence Interval, *GI* gastrointestinal system, *GU* genitourinary system, *MSK* musculoskeletal system

**Table 4:** Adjusted Odds and Hazard Ratios for sub-diagnoses in the highest three main diagnostic groups across overall, cervical, blepharospasm and tremor dystonia cohorts

Dystonia group	Pre-dystonia diagnosis (adjusted OR)				Post-dystonia diagnosis (adjusted HR)			
	Diagnosis		aOR (95% CI)	*p* value	Diagnosis		aHR (95% CI)	*p* value
A. Overall	MSK	Vertebral column syndromes	2.0 (2.03–2.14)	< 2e−16	MSK	Musculoskeletal and connective tissue diseases NOS	2.91 (2.12–4.01)	4.50E−11
		Rheumatism, excluding the back	1.82 (1.77–1.87)	< 2e−16		Vertebral column syndromes	1.97 (1.93–2.01)	< 2e−16
		Osteopathy/chondropathy/acquired musculoskeletal deformity	1.74 (1.64–1.84)	< 2e−16		Rheumatism, excluding the back	1.67 (1.64–1.7)	< 2e−16
	Resp	Lung disease due to external agents	2.37 (1.56–3.55)	3.78e−05	Resp	Lung disease due to external agents	1.74 (1.29–2.35)	0.000279
		Acute respiratory infections	1.86 (1.82–1.9)	< 2e−16		Pneumonia and influenza	1.72 (1.65–1.79)	< 2e−16
		Pneumonia and influenza	1.75 (1.65–1.85)	< 2e−16		Acute respiratory infections	1.65 (1.62–1.68)	< 2e−16
	GI	Oesophagus, stomach, and duodenal diseases	1.85 (1.78–1.92)	< 2e−16	GI	Oral cavity, salivary glands, and jaw diseases	1.69 (1.63–1.75)	< 2e−16
		Oral cavity, salivary glands, and jaw diseases	1.76 (1.68–1.84)	< 2e−16		Oesophagus, stomach and duodenal diseases	1.64 (1.59–1.69)	< 2e−16
		Other diseases of the intestines and peritoneum	1.76 (1.69–1.83)	< 2e−16		Other specified diseases of digestive system	1.62 (1.25–2.11)	0.000254
B. Cervical	Resp	Other upper respiratory tract diseases	1.7 (1.62–1.77)	2.00e−16	MSK	Musculoskeletal and connective tissue diseases NOS	3.43 (2.45–4.78)	4.54e−11
		Pneumonia and influenza	1.66 (1.55–1.76)	2.00e−16		Vertebral column syndromes	2.04 (1.99–2.09)	2.00e−16
		Chronic obstructive pulmonary disease	1.46 (1.4–1.52)	2.00e−16		Rheumatism, excluding the back	1.59 (1.56–1.63)	2.00e−16
	Infectious	Poliomyelitis & other non-arthropod-borne diseases	3.02 (1.61–5.44)	0.000343	Resp	Acute respiratory infections	1.71 (1.68–1.75)	2.00e−16
		Other infectious and parasitic diseases	1.88 (1.74–2.02)	2.00e−16		Pneumonia and influenza	1.66 (1.59–1.74)	2.00e−16
		Intestinal infectious diseases	1.85 (1.72–1.98)	2.0e−16		Other upper respiratory tract diseases	1.49 (1.43–1.54)	2.00e−16
	MSK	Vertebral column syndromes	2.04 (1.97–2.11)	2.00e−16	Infectious	Intestinal infectious diseases	1.84 (1.73–1.95)	2.00e−16
		Musculoskeletal and connective tissue diseases NOS	1.8 (1.16–2.72)	7.77e−03		Other infectious and parasitic diseases	1.6 (1.49–1.72)	2.00e−16
		Rheumatism, excluding the back	1.6 (1.55–1.65)	2.00e−16		Mycoses	1.51 (1.47–1.55)	2.00e−16
C.BSP	Neuro	Other central nervous system disorders	1.87 (1.38–2.46)	1.94e−05	Neuro	Peripheral nervous system disorders	3.12 (2.49–3.87)	2.00e−16
		Peripheral nervous system disorders	4.98 (3.87–6.3)	< 2e−16		Disorders of eye and adnexa	1.96 (1.72–2.22)	2.00e−16
		Disorders of eye and adnexa	2.73 (2.4–3.11)	< 2e−16		Diseases of the ear and mastoid process	1.54 (1.37–1.74)	2.02e−12
	MSK	Other specified diseases of musculoskeletal or connective tissue	3.15 (1.84–5.01)	6.35e−06	MSK	Osteopathies, chondropathies and acquired musculoskeletal deformities	1.79 (1.42–2.25)	8.59e−07
		Rheumatism, excluding the back	2.31 (2.03–2.63)	2.00e−16		Vertebral column syndromes	1.73 (1.52–1.95)	2.00e−16
		Osteopathies, chondropathies, and acquired musculoskeletal deformities	1.93 (1.42–2.56)	1.07e−05		Arthropathies and related disorders	1.73 (1.5–1.99)	1.92e−14
	Skin	Skin and subcutaneous tissue infections	1.35 (1.1–1.61)	9.22e−06	GI	Oesophageal, stomach and duodenal diseases	2.05 (1.76–2.4)	2.00e−16
		Other skin and subcutaneous tissue inflammatory conditions	1.69 (1.45–1.96)	1.14e−11		Hernia of abdominal cavity	1.93 (1.47–2.54)	2.70e−06
		Other skin and subcutaneous tissue disorders	1.94 (1.68–2.22)	< 2e−16		Liver, biliary, pancreas + gastrointestinal diseases NEC	1.69 (1.27–2.25)	3.18e−04
D. Tremor	Circulation	Other specified diseases of circulatory system	7.96 (2.67–21.6)	7.24e−05	GI	Other specified diseases of digestive system	2.95 (2.04–4.24)	6.69e−09
		Cerebrovascular disease	3.54 (3.13–3.99)	< 2e−16		Liver, biliary, pancreas + gastrointestinal diseases NEC	2.44 (2.25–2.64)	2.00e−16
		Other forms of heart disease	3.09 (2.84–3.37)	< 2e−16		Hernia of abdominal cavity	2.21 (2.02–2.41)	2.00e−16
	Endocrine	Disorders of thyroid gland	2.5 (2.28–2.73)	< 2e−16	MSK	Other specified diseases of musculoskeletal or connective tissue	2.36 (2.08–2.68)	2.00e−16
		Other endocrine gland diseases	2.06 (1.88–2.24)	< 2e−16		Osteopathies, chondropathies and acquired musculoskeletal deformities	2.08 (1.93–2.23)	2.00e−16
		Nutritional deficiencies	2.29 (1.93–2.7)	< 2e−16		Rheumatism, excluding the back	1.94 (1.87–2)	2.00e−16
	MSK	Arthropathies and related disorders	2.41 (2.3–2.52)	2.00e−16	Neuro	Other specified diseases of nervous system or sense organ	2.54 (2.14–3.01)	2.00e−16
		Other specified diseases of musculoskeletal or connective tissue	2.41 (2.02–2.87)	2.00e−16		Other central nervous system disorders	1.93 (1.79–2.07)	2.00e−16
		Osteopathies, chondropathies, and acquired musculoskeletal deformities	2.38 (2.19–2.58)	2.00e−16		Peripheral nervous system disorders	1.84 (1.68–2.02)	2.00e−16

*aHR* adjusted hazard ratio, *aOR* adjusted odds ratio, *95% CI* 95% Confidence Interval, *BSP* Blepharospasm, *GI* gastrointestinal system, *MSK* musculoskeletal system, *Resp* respiratory system

### Tremor

Less consistency across was observed within the tremor cohort (*n* = 15,257) with circulatory [aOR: 2.48, (95% CI 2.4–2.59)], endocrinological [aOR: 2.32, (95% CI 2.2–2.45)], and musculoskeletal [aOR: 2.25, (95% CI 2.17–2.33)] disorders, demonstrating the highest levels of association pre-dystonia diagnosis. By contrast, gastrointestinal [aHR: 2.04, (95% CI 1.97–2.12)], musculoskeletal [aHR: 1.89, (95% CI 1.83–1.95)], and nervous system [aHR: 1.73, (95% CI 1.67–1.78)] disorders were most commonly observed post-diagnosis (Table 3). Once again, the risk of developing these disorders was higher pre-dystonia diagnosis, with more specific diagnoses including disorders of the circulatory system [aOR: 7.96, (95% CI 2.67–21.6)], cerebrovascular disease [aOR: 3.54, (95% CI 3.13–3.99)], and other forms of heart disease [aOR: 3.54, (95% CI 3.13–3.99)]. Post-dystonia diagnosis, disorders of the digestive system [aHR: 2.95 (95% CI 2.04–4.24)], including liver, biliary, pancreas, and gastrointestinal disorders [aHR: 2.44, (95% CI 2.25–2.64)], had the highest association with dystonic tremor (Table 4D).

## Discussion

This study represents the first to systematically examine for the potential risk of other medical disorders, outside of psychiatric diagnoses, within a dystonia cohort using a longitudinal, linked clinical databank. As has been undertaken in the previous studies, application of the ≥ 10% prevalence rate to primary ICD-10 diagnoses enabled this work to focus on disorders with greatest clinical relevance [20], with disorders involving musculoskeletal, respiratory, and nervous system consistently observed across the dystonia diagnostic cohorts. Subsequent calculation of adjusted OR and HR, pre- and post-dystonia diagnosis, respectively, identified higher associations with infectious disorders in cervical dystonia, cutaneous and GI disorders in blepharospasm, and circulatory and GI disorders with dystonic tremor. Analysis of the more specific individual secondary diagnoses identified circulatory system disorders, cerebrovascular disease, and disorders of the digestive system in dystonic tremor, peripheral nervous system and other musculoskeletal or connective tissue disorders with blepharospasm, and vertebral column syndromes in cervical dystonia.

Analysis of adjusted OR and HR consistently observed musculoskeletal disorders to be one of the most common diagnoses to be associated with dystonia. Higher rates of musculoskeletal diagnoses have been observed for some time in dystonia [21], with recognition that initial clinical presentation may be via musculoskeletal and orthopaedic services, potentially contributing to the delay in dystonia diagnosis [22, 23]. Much of the reported literature to date has highlighted this higher burden of musculoskeletal difficulties in those diagnosed with cervical dystonia, identifying disorders such as vertebral subluxation, fractures, and spinal degenerative changes [24, 25]. These are coupled with risk of secondary complications such as the vertebral column changes observed in the cervical dystonia cohort in this study post-dystonia diagnosis, with more severe complications such as spinal cord compression noted to be of higher risk amongst those with other forms of bone disease such as osteoporosis and rheumatoid arthritis [25, 26]. A previous Finnish national registry study focusing on co-morbidity in those diagnosed with cervical dystonia found odds ratios for cervical intervertebral disc disorders [OR 5.9, (95% CI 3.1–1.1)] to be the highest for any diagnosis outside of psychiatric symptoms, with shoulder [OR 2.26, (95% CI 1.2–4.4)] and intervertebral disc disorders [OR 1.41, (95% CI 0.7–2.7)] being the two most common physical causes for retirement in those with cervical dystonia [12].

Respiratory disorders have been previously linked with dystonia, primarily in conjunction with cranio-cervical dystonias and predominantly involving the upper airways, typically in the context of laryngeal, platysmal, and paranasal muscular involvement [10]. In relation to infective illness, this has been predominantly described in paediatric dystonia cohorts, primarily in the form of Grisel’s syndrome [27], although more recently dystonia has been described in the context of sequelae to COVID-19 infection [28]. Disorders associated with the peripheral nervous system were associated at higher rates in the blepharospasm and dystonic tremor groups. This may in part relate to the sensory disturbances well described in dystonic cohorts, and in particular sensory discrimination thresholds as endophenotypes in genetic dystonia cohorts [29]. Significant increases to sensory discrimination thresholds have also been more specifically described in dystonia tremor cohorts, distinct from essential tremor, as well as those cranial forms of dystonia, including blepharospasm [30, 31]. More recent work involving those diagnosed with blepharospasm has also found a reduction in activity of brainstem inhibitory interneurons following stimulation of both the supraorbital nerve and upper limb peripheral nerves [32]. Additional neurological diagnoses identified in the blepharospasm cohort included those involving the ear and mastoid processes. Although not frequently reported, a few studies have described hearing loss in dystonia, with 50% of a single *GCH1* mutation-positive cohort reported to experience symptoms of hearing loss, as well as there being the rare but recognised X-linked dystonia deafness syndrome [33, 34].

Finally, GI disturbance was identified in the overall, blepharospasm and dystonic tremor cohorts, and more specifically involvement of the upper GI tract and digestive system. There are few case reports or case series highlighting these symptoms; however, a recent analysis of the gut microbiome in a dystonia cohort of (27 cervical dystonia, 20 dopa-responsive dystonia, and 24 myoclonus dystonia) identified changes to the gut flora, compared to controls, with this linked to non-motor symptom severity and plasma neurotransmitter levels. In addition, several metabolic pathways linked with the nervous system, notably tryptophan degradation, were present at lower levels in the dystonia cohort [35]. By contrast, functional analysis in a distinct cohort of individuals diagnosed with isolated dystonia identified genes related to tryptophan and purine biosynthesis to be at higher levels in the gut microbiota, compared to controls, while serum metabolomic analysis found altered levels of l-glutamic acid, taurine, and d-tyrosine, again linking to changes in neurotransmitter metabolism [36].

Although this study provides a robust examination of potential co-morbid medical diagnoses across a spectrum of idiopathic dystonia diagnoses, this work is limited primarily by clinical detail being constrained to diagnostic codes, with no further specific symptomatic detail that may allow for greater understanding of the nature, variability, and severity of the associated phenotypes. In addition, we have used only primary care diagnostic codes, leading to the potential risk of coding errors or diagnoses being missed. Although primary care diagnostic codes reflect those entered in the primary care setting, in the instance of more specialised diagnoses, such as dystonia, this is information is typically entered after correspondence from secondary or tertiary specialist care services. In addition, delays in dystonia diagnosis are well recognised, which may have impacted our analyses, particularly in determining those diagnoses identified at being at increased rates prior to dystonia diagnosis. Finally, although, during our analyses, we have corrected for the number of individual clinical contacts across both primary and secondary care sectors, there remains potential for referral bias which too may be reflected in the diagnoses identified both pre- and post-dystonia diagnosis.

In summary, this study represents the first to systematically undertake a longitudinal evaluation of the rate and type of co-morbid clinical diagnoses in idiopathic dystonia, consistently demonstrating higher rates of respiratory, musculoskeletal, and nervous system disorders, with higher prevalence of rates prior to dystonia diagnosis rather than in subsequent years. This work highlights the need for greater awareness of these disorders in the context of dystonia, with specific evaluation to ensure identification and appropriate management. Recognition of these increased rates of co-morbidity also represents an important component of any future care pathways designed for the comprehensive management of idiopathic dystonia.

### Supplementary Information

Below is the link to the electronic supplementary material.

Supplementary file1 (DOCX 33 KB)

## Data Availability

The data used in this study is available from the Secure Anonymised Information Linkage (SAIL) Databank at Swansea University, Swansea, UK, which is part ofthe national e-health records research infrastructure for Wales. All proposals to use SAIL datasets must comply with HIRU’s information governance policy and are subject to review by an independent Information Governance Review Panel (IGRP). Before data can be accessed, approval must be given by the IGRP. Requests to access these datasets should be directed to www.saildatabank.com/application-process.
