# Supplementary material for: Prevalence and temporal relationship of clinical co-morbidities in idiopathic dystonia: a UK linkage-based study
Source: J Neurol. 2024 Mar 21;271(6):3398–408. doi: 10.1007/s00415-024-12284-6 (PMC11136734; doi:10.1007/s00415-024-12284-6)
Supplement: Supplementary file 1 — Supplementary file1 (DOCX 33 KB) [file 415_2024_12284_MOESM1_ESM.docx]

**Supplementary Table 1: Read codes used to identify diagnostic sub-categories.**

| **Diagnosis** | | **Read Code** |
| --- | --- | --- |
| **Infectious and Parasitic disease (A)** | Intestinal infectious diseases | A0 |
|  | Poliomyelitis & other non-arthropod borne diseases | A4 |
|  | Mycoses | AC |
|  | Other infectious and parasitic diseases | AD |
| **Endocrine, nutritional, metabolic and immunity disorders (C)** | Disorders of thyroid gland | C0 |
|  | Other endocrine gland diseases | C1 |
|  | Nutritional deficiencies | C2 |
| **Nervous system and sense organ diseases (F)** | Other central nervous system disorders | F2 |
|  | Peripheral nervous system disorders | F3 |
|  | Disorders of eye and adnexa | F4 |
|  | Diseases of the ear and mastoid process | F5 |
|  | Other specified diseases of nervous system or sense organ | Fy |
| **Respiratory system diseases (H)** | Acute respiratory infections | H0 |
|  | Pneumonia and influenza | H2 |
|  | Chronic obstructive pulmonary disease | H3 |
|  | Lung disease due to external agents | H4 |
| **Circulatory system diseases (G)** | Other forms of heart disease | G5 |
|  | Cerebrovascular disease | G6 |
|  | Other specified diseases of circulatory system | Gy |
| **Digestive system diseases (J)** | Oral cavity, salivary glands, and jaw diseases | J0 |
|  | Oesophagus, stomach and duodenal diseases | J1 |
|  | Hernia of abdominal cavity | J3 |
|  | Other diseases of the intestines and peritoneum | J5 |
|  | Liver, biliary, pancreas & gastrointestinal diseases NEC | J6 |
|  | Other specified diseases of digestive system | Jy |
| **Skin and subcutaneous tissue diseases (M)** | Skin and subcutaneous tissue infections | M0 |
|  | Other skin and subcutaneous tissue inflammatory conditions | M1 |
|  | Other skin and subcutaneous tissue disorders | M2 |
| **Musculoskeletal and connective tissue diseases (N)** | Arthropathies and related disorders | N0 |
|  | Vertebral column syndromes | N1 |
|  | Rheumatism, excluding the back | N2 |
|  | Osteopathies, chondropathies and acquired musculoskeletal deformities | N3 |
|  | Other specified diseases of musculoskeletal or connective tissue | Ny |
|  | Musculoskeletal and connective tissue diseases NOS | Nz |

**Supplementary Table 2: Demographic Characteristics of idiopathic dystonia and control cohorts**

|  | **Dystonia (n, %)** | **Cervical Dystonia (n, %)** | **Blepharospasm (n, %)** | **Tremor (n, %)** |
| --- | --- | --- | --- | --- |
| Total | 54,166 | 36,846 | 1,291 | 15,257 |
| Male | 21,712 (40.1) | 14,621 (39.7) | 482 (37.3) | 6300 (41.3) |
| Female | 32,454 (59.9) | 22,225 (60.3) | 809 (62.7) | 8957 (58.7) |
| Age at dystonia diagnosis (median, IQR) | 34 (32) | 31 (27) | 45 (31) | 48 (41) |

**Supplementary Table 3. Frequency of comorbid diagnoses within major categories amongst individuals with Cervical Dystonia**

|  | **Total number of cases** | | **Pre-dystonia diagnosis** | | **Post-dystonia diagnosis** | |
| --- | --- | --- | --- | --- | --- | --- |
| **Disease category** | **Cervical Dystonia (n, %)**  **(n = 36,846)** | **Controls**  **(n, %)**  **(n = 216,574)** | **Cervical Dystonia (n, %)**  **(n = 36,846)** | **Controls**  **(n, %)**  **(n = 216,574)** | **Cervical Dystonia (n, %)**  **(n = 36,846)** | **Controls**  **(n, %)**  **(n = 216,574)** |
| Infectious and parasitic diseases | **19,209 (52.1)** | **77,601 (35.8)** | **9293 (25.2)** | **34,845 (16.09)** | **14,709 (39.9)** | **56,649 (26.2)** |
| Neoplasms | **6647 (18.0)** | **32,829 (15.2)** | 2337 (6.3) | 11,599 (5.36) | **5030 (13.7)** | **24,509 (11.3)** |
| Endocrine, nutritional, metabolic and immunity disorders | **5345 (14.5)** | **27,479 (12.7)** | 1982 (5.4) | 10,654 (4.92) | **4116 (11.2)** | 20,536 (9.5) |
| Diseases of blood and blood-forming organs | 2175 (5.9) | 10,642 (4.9) | 823 (2.2) | 3832 (1.77) | 1516 (4.1) | 7477 (3.45) |
| Nervous system and sense organ diseases | **22,304 (60.5)** | **97,175 (44.9)** | **10,987 (29.8)** | **45, 136 (20.84)** | **17,997 (48.8)** | **75,086 (34.7)** |
| Circulatory system diseases | **8448 (22.9)** | **45,537 (21)** | 3471 (9.4) | 19,852 (9.17) | **6521 (17.7)** | **33,692 (15.6)** |
| Respiratory system diseases | **27,542 (74.7)** | **121,244 (56)** | **16,208 (44)** | **62,815 (29)** | **23,566 (64)** | **98,051 (45.3)** |
| Digestive system diseases | **15,134 (41.1)** | **62,858 (29)** | **6338 (17.2)** | **25,922 (11.97)** | **11,899 (32.3)** | **47,280 (21.8)** |
| Genitourinary system diseases | **16,822 (45.7)** | **73,045 (33.7)** | **7613 (20.7)** | **31,181 (14.4)** | **13,706 (37.2)** | **57,020 (26.3)** |
| Complications of pregnancy, childbirth, and the puerperium | 3657 (9.9) | 16333 (7.5) | 1486 (4) | 6587 (3.04) | 2558 (6.9) | 11,409 (5.3) |
| Skin and subcutaneous tissue diseases | **23,914 (64.9)** | **108,202 (50)** | **11,428 (31)** | **48,359 (22.33)** | **20,084 (54.5)** | **87,306 (40.3)** |
| Musculoskeletal and connective tissue | **24,298 (65.9)** | **101,594 (46.9)** | **11,566 (31.4)** | **44,933 (20.75)** | **21,407 (58.1)** | **84,362 (39)** |
| Congenital anomalies | 1126 (3.1) | 3949 (1.8) | 489 (1.3) | 1693 (0.78) | 693 (1.9) | 2424 (1.1) |
| Perinatal conditions | * | 639 (0.3) | 79 (0.2) | 288 (0.13) | 89 (0.2) | 354 (0.2) |

**Legend:** Bold values represent >10% affected, * masked to prevent disclosure of numbers <5, Note, the pre- and post- numbers do not add up to the total where individuals have a diagnosis before and after a dystonia diagnosis

**Supplementary Table 4. Frequency of comorbid diagnoses within major categories amongst individuals with Blepharospasm**

|  | **Total number of cases** | | **Pre-dystonia diagnosis** | | **Post-dystonia diagnosis** | |
| --- | --- | --- | --- | --- | --- | --- |
| **Disease category** | **Blepharospasm**  **(n, %)**  **(n = 1291)** | **Controls**  **(n, %)**  **(n = 216,574)** | **Blepharospasm (n, %)**  **(n = 1291)** | **Controls**  **(n, %)**  **(n = 216,574)** | **Blepharospasm**  **(n, %)**  **(n = 1291)** | **Controls**  **(n, %)**  **(n = 216,574)** |
| Infectious and parasitic diseases | **553 (42.8)** | **77,601 (35.8)** | **296 (23.5)** | **34,845 (16.09)** | **397 (31.5)** | **56,649 (26.2)** |
| Neoplasms | **293 (22.7)** | **32,829 (15.2)** | 124 (9.8) | 11,599 (5.36) | **212 (16.8)** | **24,509 (11.3)** |
| Endocrine, nutritional, metabolic and immunity disorders | **229 (17.7)** | **27,479 (12.7)** | 101 (8) | 10,654 (4.92) | **162 (12.8)** | 20,536 (9.5) |
| Diseases of blood and blood-forming organs | 87 (6.7) | 10,642 (4.9) | 32 (2.5) | 3832 (1.77) | 60 (4.8) | 7477 (3.45) |
| Nervous system and sense organ diseases | **857 (66.4)** | **97,175 (44.9)** | **503 (39.9)** | **45, 136 (20.84)** | **677 (53.6)** | **75,086 (34.7)** |
| Circulatory system diseases | **431 (33.4)** | **45,537 (21)** | **203 (16.1)** | 19,852 (9.17) | **321 (25.4)** | **33,692 (15.6)** |
| Respiratory system diseases | **871 (67.5)** | **121,244 (56)** | **544 (43.1)** | **62,815 (29)** | **699 (55.4)** | **98,051 (45.3)** |
| Digestive system diseases | **567 (43.9)** | **62,858 (29)** | **265 (21)** | **25,922 (11.97)** | **441 (34.9)** | **47,280 (21.8)** |
| Genitourinary system diseases | **580 (44.9)** | **73,045 (33.7)** | **290 (23)** | **31,181 (14.4)** | **442 (35)** | **57,020 (26.3)** |
| Complications of pregnancy, childbirth, and the puerperium | * | 16333 (7.5) | 31 (2.5) | 6587 (3.04) | 41 (3.2) | 11,409 (5.3) |
| Skin and subcutaneous tissue diseases | **837 (64.8)** | **108,202 (50)** | **465 (36.8)** | **48,359 (22.33)** | **664 (52.6)** | **87,306 (40.3)** |
| Musculoskeletal and connective tissue | **857 (66.4)** | **101,594 (46.9)** | **475(37.6)** | **44,933 (20.75)** | **729 (57.8)** | **84,362 (39)** |
| Congenital anomalies | * | 3949 (1.8) | 17 (1.3) | 1693 (0.78) | 23 (1.8) | 2424 (1.1) |
| Perinatal conditions | ***** | 639 (0.3) | 0 (0) | 288 (0.13) | 0 (0) | 354 (0.2) |

**Legend:** Bold values represent >10% affected, * masked to prevent disclosure of numbers <5, Note, the pre- and post- numbers do not add up to the total where individuals have a diagnosis before and after a dystonia diagnosis

**Supplementary Table 5. Frequency of comorbid diagnoses within major categories amongst individuals with dystonic tremor**

|  | Total number of cases | | Pre-dystonia diagnosis | | Post-dystonia diagnosis | |
| --- | --- | --- | --- | --- | --- | --- |
| Disease category | Tremor (n, %)  (n = 15,257) | Controls (n, %)  (n = 216,574) | Tremor (n, %)  (n = 15,257) | Controls (n, %)  (n = 216,574) | Tremor (n, %)  (n = 15,257) | Controls (n, %)  (n = 216,574) |
| Infectious and parasitic diseases | 6734 (44.1) | 77,601 (35.8) | 3484 (24.4) | 34,845 (16.09) | 4706 (32.9) | 56,649 (26.2) |
| Neoplasms | 3223 (21.1) | 32,829 (15.2) | 1500 (10.5) | 11,599 (5.36) | 2184 (15.3) | 24,509 (11.3) |
| Endocrine, nutritional, metabolic and immunity disorders | 3776 (24.7) | 27,479 (12.7) | 1770 (12.4) | 10,654 (4.92) | 2693 (18.8) | 20,536 (9.5) |
| Diseases of blood and blood-forming organs | 1375 (9.0) | 10,642 (4.9) | 549 (3.8) | 3832 (1.77) | 932 (6.5) | 7477 (3.45) |
| Nervous system and sense organ diseases | 8989 (58.9) | 97,175 (44.9) | 5093 (35.6) | 45, 136 (20.84) | 6800 (47.5) | 75,086 (34.7) |
| Circulatory system diseases | 5725 (37.5) | 45,537 (21) | 3282 (22.9) | 19,852 (9.17) | 4018 (28.1) | 33,692 (15.6) |
| Respiratory system diseases | 10382 (68.0) | 121,244 (56) | 6501 (45.4) | 62,815 (29) | 8335 (58.3) | 98,051 (45.3) |
| Digestive system diseases | 5725 (37.5) | 62,858 (29) | 3755 (26.2) | 25,922 (11.97) | 5265(36.8) | 47,280 (21.8) |
| Genitourinary system diseases | 6715 (44.0) | 73,045 (33.7) | 3525 (24.6) | 31,181 (14.4) | 5018 (35.1) | 57,020 (26.3) |
| Complications of pregnancy, childbirth, and the puerperium | 791 (5.2) | 16333 (7.5) | 308 (2.2) | 6587 (3.04) | 559 (3.9) | 11,409 (5.3) |
| Skin and subcutaneous tissue diseases | 9522 (62.4) | 108,202 (50) | 5220 (36.5) | 48,359 (22.33) | 7506 (52.5) | 87,306 (40.3) |
| Musculoskeletal and connective tissue | 10124 (66.4) | 101,594 (46.9) | 6106 (42.7) | 44,933 (20.75) | 8202 (57.3) | 84,362 (39) |
| Congenital anomalies | 397 (2.6) | 3949 (1.8) | 191 (1.3) | 1693 (0.78) | 216 (1.5) | 2424 (1.1) |
| Perinatal conditions | 54 (0.4) | 639 (0.3) | 28 (0.2) | 288 (0.13) | 26 (0.2) | 354 (0.2) |

**Legend:** Bold values represent >10% affected, * masked to prevent disclosure of numbers <5, Note, the pre- and post- numbers do not add up to the total where individuals have a diagnosis before and after a dystonia diagnosis
